# Engineering Considerations to Produce Bioactive Compounds from Plant Cell Suspension Culture in Bioreactors

**DOI:** 10.3390/plants10122762

**Published:** 2021-12-14

**Authors:** Elizabeth Alejandra Motolinía-Alcántara, Carlos Omar Castillo-Araiza, Mario Rodríguez-Monroy, Angélica Román-Guerrero, Francisco Cruz-Sosa

**Affiliations:** 1 Departamento de Biotecnología, Universidad Autónoma Metropolitana-Iztapalapa, Av. Ferrocarril de San Rafael Atlixco 186, Ciudad de México 09310, Mexico; eama@xanum.uam.mx; 2 Departamento de Ingeniería de Procesos e Hidráulica, Universidad Autónoma Metropolitana-Iztapalapa, Av. Ferrocarril de San Rafael Atlixco 186, Ciudad de México 09310, Mexico; coca@xanum.uam.mx; 3 Centro de Desarrollo de Productos Bióticos (CEPROBI), Departamento de Biotecnología, Instituto Politécnico Nacional (IPN), Yautepec 62731, Mexico; mrmonroy@ipn.mx

**Keywords:** medicinal plant, bioactive compounds, plant-derived secondary metabolites (PDSM), cell suspension culture (CSC), bioreactor engineering

## Abstract

The large-scale production of plant-derived secondary metabolites (PDSM) in bioreactors to meet the increasing demand for bioactive compounds for the treatment and prevention of degenerative diseases is nowadays considered an engineering challenge due to the large number of operational factors that need to be considered during their design and scale-up. The plant cell suspension culture (CSC) has presented numerous benefits over other technologies, such as the conventional whole-plant extraction, not only for avoiding the overexploitation of plant species, but also for achieving better yields and having excellent scaling-up attributes. The selection of the bioreactor configuration depends on intrinsic cell culture properties and engineering considerations related to the effect of operating conditions on thermodynamics, kinetics, and transport phenomena, which together are essential for accomplishing the large-scale production of PDSM. To this end, this review, firstly, provides a comprehensive appraisement of PDSM, essentially those with demonstrated importance and utilization in pharmaceutical industries. Then, special attention is given to PDSM obtained out of CSC. Finally, engineering aspects related to the bioreactor configuration for CSC stating the effect of the operating conditions on kinetics and transport phenomena and, hence, on the cell viability and production of PDSM are presented accordingly. The engineering analysis of the reviewed bioreactor configurations for CSC will pave the way for future research focused on their scaling up, to produce high value-added PDSM.

## 1. Introduction

The plant world has been exploited for thousands of years by human cultures for preventing pain, producing pleasure, usage in religious ceremonies, and treating different diseases and illnesses. Recently, the World Health Organization (WHO) estimated that 80 percent of people worldwide rely on herbal medicines for some aspect of their primary healthcare importance. Medicinal plants have great significance in the health industry. Today, almost 25% of modern medicines are obtained or derived from natural sources [1,2,3]. To this end, according to a recent report by the British Broadcasting Corporation (BBC), plant-derived drugs will grow from USD 29.3 billion in 2017 to around USD 39.2 billion by 2022, with a compound annual growth rate (CAGR) of 6.1% per year for the same period.

The chemical entities responsible for the biological activity in medicinal plants are organic molecules classified as secondary metabolites. Although there are more than 50,000 plant species used for medicinal purposes, bioactive compounds in plant tissues generally account for less than 1% (wt.), leading to the overexploitation, threatening, or extinction of vegetal species [4,5,6]. In agreement with Süntar et al. [7], half of the drugs used for clinical treatments are obtained out of natural sources, accounting for 185 chemical compounds approved by the FDA [8]. Due to the growing interest in obtaining phytochemicals from plant-derived secondary metabolites (PDSM), the use of biotechnologies based on the plant cell culture (PCC) results in a promising, sustainable, and environmentally friendly way of overcoming the problems related to either the traditional cultivation of medicinal plants (including variations in crop quality associated with drought or flood crops, diseases or pest attacks on plants, or the chemical synthesis of bioactive compounds, allowing a better control in the quality and higher yields of the desired products to be achieved.

Academia and industry, nowadays, aim their research on PCC for large-scale production of bioactive compounds by using callus cells, immobilized cells, embryos, roots, shoots, and suspended cells [9,10]. Among PCC, the plant cell suspension culture (CSC) is one of the processes that presents excellent scaling-up attributes for producing plant-derived secondary metabolites (PDSM). Nevertheless, although the use of CSC seems an attractive bioreaction concept for large-scale production of bioactive compounds [11,12,13], there is a lack of information related to the engineering of the bioreactor technology because of the complex interaction between the operational variables and its performance regarding microbiology, thermodynamics, kinetics, and transport phenomena, which, in turn, impact on the production and quality of PDSM.

This review, in its first part, states the main bioactive compounds found in plants with great commercial relevance, giving an overview of the types of compounds and their different biological activities, and stressing the relevance of PCC for satisfying the today’s demand for bioactive compounds. The second part is focused on the production of bioactive compounds by CSC, summarizing the engineering strategies followed in the literature for achieving large-scale production and indicating the characteristics required from plant tissues to obtain CSC with adequate properties. Bioreactor configurations implemented for CSC are also described in this section. Because of its impact on the design and scale-up of CSC, the third part elucidates the impact of the bioreactor configuration and operational conditions on the performance of plant cell cultures during the production of PDSM.

## 2. Bioactive Compounds in Plants

Medicinal plants are considered as a resource for bioactive compounds that can be used for obtaining pharmacopeial or non-pharmacopeial drugs, contributing to the rising interest and demand for plant-derived phytochemicals [14,15,16]. Their main activities include antioxidant, anti-inflammatory, antiproliferative, anticancer, anti-neurodegenerative disorders, and chronic diseases, among other health properties [17]. These bioactive compounds are considered as plant-derived secondary metabolites (PDSM) since, in contrast to the primary metabolites, they do not play an active role in the direct metabolic process or growth of the plant, but their importance relies on their interactions with biotic and abiotic stresses in defense of the plant viability [7,18,19].

**Figure 1:**
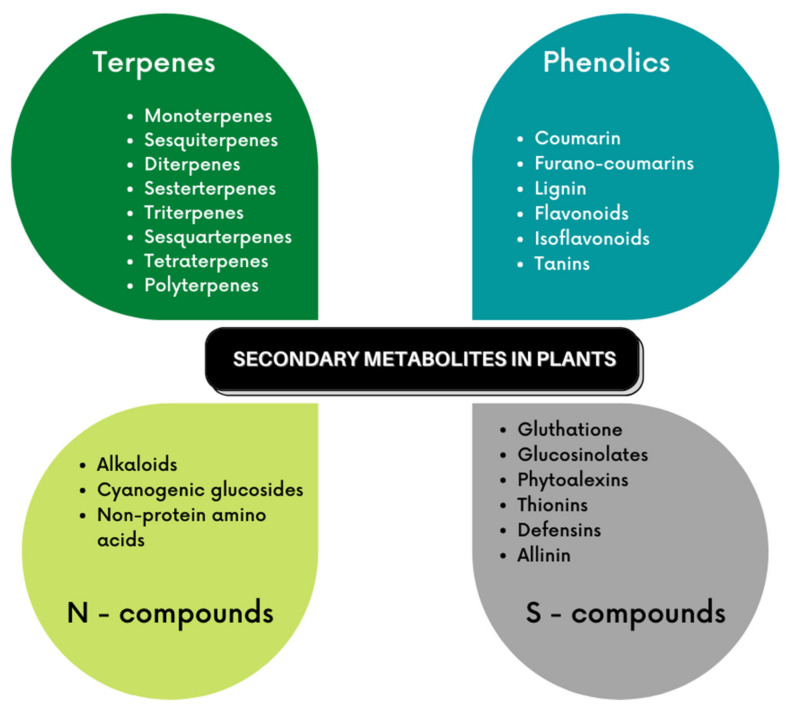
Classification and types of PDSM.

PDSM are classified in three main groups: (i) terpenes and terpenoid compounds formed by 5-C isopentanoid units, (ii) phenolic compounds derived from the shikimic acid pathway, and (iii) nitrogen and sulfur comprising compounds (Figure 1) synthesized from common amino acids [18]. Their classification is based on their composition, structure, the pathway used for their biosynthesis, and their chemical nature (solubility, polarity, reactivity) [17,20,21,22]. PSDM are normally produced out of specific plant species or taxonomy [18]. Table 1 describes the main characteristics and biological activities reported for the three groups of PDSM.

PDSM are, in general, extracted from roots and aerial parts of the plant, with typical yields below 1% wt. Their production usually takes very long cultivation times for reaching the plant maturity and ensuring the highest PDSM production, thus requiring large amounts of plant material for satisfying the demand of bioactive compounds in the food and pharmaceutical industries, and leading to the overexploitation and extinction of the vegetal species, despite the risk of unpredictable differences in the homogeneity and quality of the extracted vegetal due to environmental factors during cultivation [16,18,23,24]. Therefore, it is important to implement novel technologies that allow the production of bioactive compounds in more sustainable and better controlled processes. Among the biotechnological strategies proposed in the literature to produce PDSM out of PCC, the root culture, shoot culture, hairy root culture, and cell suspension culture are some of the most studied in the laboratory scale.

**Table 1:** Characteristics and biological activities reported for PDSM in medicinal plants.

Compound	Characteristics	Representative Compounds	Biological/Pharmacological Properties	References
Alkaloids	Complex organic molecules with a heterocyclic nitrogen ring. ↑ 10,000 compounds isolated ↑ 300 families of plants	Piperine; nicotine, vasicine; theobromine; caffeine; aconitine; atropine; quinine; capsaicin, ephedrine; paclitaxel; morphine; berberine; vincristine;	Chemotherapeutic agents; antiproliferative; antimicrobial and antiparasitic activities; analgesic, anti-hyperglycemic, Alzheimer’s and Parkinson’s diseases, stroke, epilepsy, fungicide.	[25,26,27,28,29]
Phenolic compounds	They are diverse in structure, and present in common the hydroxylated aromatic rings. ↑ 8000 different compounds identified ↑ 300 families of plants	*Simple phenolics*: hydroquinone, pyrogallol acid. *Single phenolic ring*: gallic acid, salicylic acid, caffeic acid, hydroxycinnamic acids. *Two phenolic rings:* Xanthones, stilbenes, flavonoids. *Quinines:* Benzoquinones, naphtaquinones anthraquinones, tannins and lignans.	Antioxidant, anti-inflammatory, anticarcinogenic, cardiovascular protective effect, antidiabetic, anti-obesity, antihemolytic, antibacterial, liver protection, antiatherogenic, antiviral, neuroprotective functions.	[30,31,32,33,34,35]
Terpenes	Synthesized from acetate via the mevalonic acid pathway. They comprise natural hydrocarbons with isoprene blocks. ↑ 23,000 compounds identified.	*Monoterpenes:* menthol. *Sesquiterpenes:* zingiberene. *Diterpenes:* retinol. *Triterpenes*: squalene. *Tetraterpene*: β-carotene.	Anti-hypertensive activity, antimicrobial, insecticide, fungicide, antioxidant, anticonvulsant, anti-tumor and neurotrophic effects, immune function, skin protection, UV protection, anticancer, antiparasitic, antihyperglycemic.	[28,29,30,36,37]
Sulfur-rich compounds (SRGs)	The structure is composed of a β-D-glucosyl residue linked by a sulfur atom. About 137 SRGs identified in plants	Glutathione, glucosinolates, phytoalexines, thionines,	Enzyme regulator, intercellular signaling molecule, antitumor effect, Burkitt lymphoma treatment, anticancer.	[38,39,40,41]

## 3. Plant Cell Culture

Plant cell culture (PCC) is nowadays recognized as a promising, renewable, sustainable, and environmentally friendly alternative to obtain PDSM out of wild plants. PCC accounts for the virtues of whole-plant cultivation systems and offers significant advantages, such as controlled manufacture due to standardized environmental conditions, i.e., it is not seasonal dependent, makes use of low amounts of water, and pesticides and herbicides are not required, achieving better quality in the desired product [42,43]. The establishment of different types of plant cell cultures are aimed for regenerating organs or somatic embryos for plant propagation, virus elimination, genetic manipulation, germplasm storage, and for in vitro production of PDSM [44,45]. The main differences found in the literature about in vitro plant cell cultures with whole wild plants comprise a higher cell growth intensity, the mechanisms of synthesis and reserve organelles for PDSM, and the concentration of PDSM. Despite most of the PDSM contents recovered in plant cell cultures usually being lower than those obtained in wild plants, there are some successful cases where super-producer strains have been achieved [46,47]. According to the literature [48], PCC is defined as the process where plant cells or plant tissues are isolated from plant organs and cultivated under artificial environmental conditions, taking advantage of plants’ regeneration ability by means of cuttings or explants. Thus, the resultant proliferated plant cells maintain the genetic information from the donor plant. In the literature [49], it has been elucidated how PCC works as a technological platform where chromosome doubling and artificial polyploidy induction are favored by getting the overexpression of key genes involved in the synthesis of PDSM and, hence, favoring the production of both high-value bioactive compounds and plants with more agronomical potency. Plant cell cultures include the production of callus and immobilized cells, embryos, roots, shoots, and cell suspensions, extending the advantages of manipulation with a better understanding about the intricate mechanisms of synthesis of PDSM [44].

### 3.1. Types of Cell Cultures

Calluses relate to the massive growth of cells and the buildup of agglomerated dedifferentiated cells, that may be able to revamp the complete plant, acquiring features like meristematic cells and developing new stem cells, which are able to form new individual plants [50]. Somatic embryos are obtained by the tissue formation from somatic cells or callus, having as the main objective the micropropagation of species seeds. Hairy roots culture is usually obtained by the infection of plant cells with *Agrobacterium rhizogenes*, resulting in the transformation of callus into differentiated tissues [48].

Even though there are several studies where the production of PDSM from callus cultures and differentiated cells/tissues are used, the cell suspension culture from dedifferentiated cells is mostly preferred [51,52]. Cell suspension cell culture (CSC) is considered as a simple and cost-effective method, allowing suitable conditions for cells to produce compounds identical to those from parental cells to be achieved, offering advantages such as setting stable systems for continuous PDSM production with homogeneity in yields and quality, as well as offering the possibility of synthesizing new compounds and greater potential for PDSM commercial application [51,53]. Therefore, CSC has been demonstrated to be the selected biotechnological tool for obtaining high-value PDSM, such as taxol [53,54], resveratrol [55,56], and ginsenosides [57], among others. To this end, further discussions will be centered on CSC for producing PDSM at laboratory and larger scales using different bioreactor configurations. Table 2 shows recent successful examples where plant cell culture is used for producing PDSM with pharmacological relevance.

### 3.2. Plant Cell Suspension Culture

Plant cell suspension culture (CSC) represents a cost-effective and simple biological process for the synthesis of PDSM at large scales [51]. This production concept takes advantage of plant cells as biosynthetically totipotent structures, being capable of obtaining bioactive compounds with identical properties to those contained in plant stem cells, offering potential advantages regarding quality and yield of PDSM [51,93]. To this end, although there are engineering challenges, CSC offers greater potential for industrial applications in large-scale bioreactors than plant tissue and organ cultures. Although the latter ones offer better genetic stability in propagated cells, the design of the bioreactors for their maintenance usually requires greater investments and careful experimentation in the preliminary scale-up stage [42,94].

**Figure 2:**
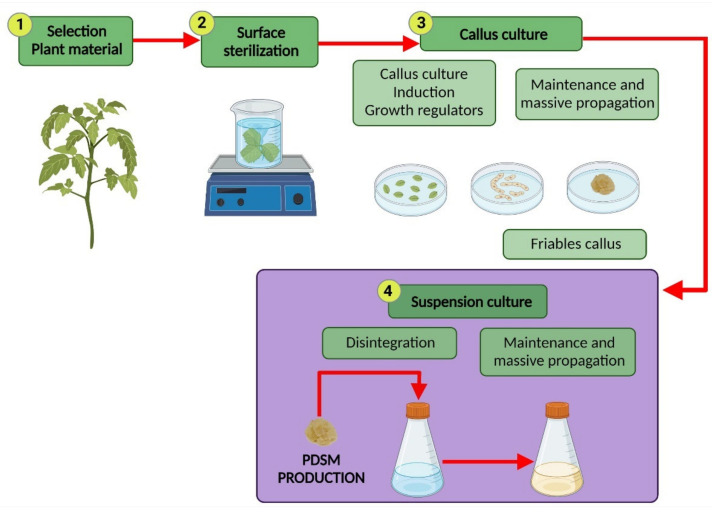
General steps for obtaining cell suspension culture. PDSM means plant-derived secondary metabolites. * Schemes were created with BioRender.com.

At a laboratory scale, CSC, in general, uses dedifferentiated plant cells and involves four essential stages as shown in Figure 2 [42].

Success in the operation of suspension cultures depends on the induction and obtention of friable callus (stage 3) through the exposure to growth regulators, such as auxins and cytokinin. The final step (stage 4) comprises the transfer and maintenance of this cell culture in a liquid. CSC may become unstable when subjected to prolonged culture times, causing differences in the quality and quantity of PDSM; this behavior is due to the consumption and reduced availability of nutrients in the culture media, in addition to genetic variations that can restrict the conservation of the high-yield cell line [9]. Among the strategies used for improving the production of PDSM in CSC is the modification in the culture media composition (different carbon, nitrogen, and phosphorous sources) for optimizing the nutrient availability during the culture time [6,9], and the use of biotic or abiotic elicitors that trigger the defense response from plant cells promoting the secondary metabolism through the introduction of chemical or physical stresses [7,51,95]. Biotic elicitors are complex compounds derived from biological sources, including plant-derived polysaccharides, such as pectin and cellulose, and microbial-derived polysaccharides, such as chitin and glucan [6,44], and plant immune-signaling molecules, such as jasmonic acid [96], salicylic acid [97,98], and methyl jasmonate [96]. Abiotic elicitors include inorganic salts, heavy metals, UV irradiation, high salinity, and pressure [99].

### 3.3. Commercial Production of PDSM from CSC

The current production of various drugs, cosmetics, and food ingredients is obtained using plant cell cultures, especially in the form of CSC, as these offer several advantages over other technologies, such as better control during the production of PDSM, a larger feasibility for the scaling up of the process, and shorter production cycles, being environmentally responsible and sustainable processes. The application of CSC to obtain commercial products dates back to the 1960s [7,9,24,100]. Table 3 shows a selection of plant cell extracts that have been successfully manufactured at a commercial scale for pharmaceutical purposes. So, by way of history, the first report about industrial manufacturing of bioactive compounds derived from CSC was found for Shikonin from *L. erythrorhizon* by Mitsui Petrochemical Ind., now Mitsui Chemicals, Inc. (Tokyo, Japan). To date, Taxol^®^, manufactured by Phyton Biotech, Inc. (Delta, BC, Canada), and Genexol, the commercial name for paclitaxel compound by Samyang Genex, represent the cancer drugs with greater demand in the market, with annual sales reaching up to 200–300 kg per year [101]. In agreement with the information available at the website for manufacturers, the production volume for PDSM increases from a few cubic meters to 75 m^3^ equivalent, to reach 880 m^3^ per year [102].

### 3.4. Typical Bioreactor Configurations

Bioreactors are defined as containers used to provide a controlled environment to transfer nutrients and oxygen to cell cultures in adequate concentrations that allow the cell to maintain its primary and secondary metabolic activity. Because plant cells, as well as other micro-organisms, are more sensitive and less stable than chemical compounds, bioreactor designs must be robust enough to provide a greater degree of control over process disturbances and contamination and achieve high productivities, high quality products, and cost effectiveness. The bioreactor design and its optimal operation depend on the determination of the operating conditions giving rise to the required product formation, minimizing the cost of the process [115]. The most common bioreactor configurations utilized for commercial and large-scale production consist in stirred tank bioreactor (STB), wave stirred bioreactor (WSB), air-lift bioreactor (ALB), and bubble column (BC). The selection of the bioreactor configuration is frequently established by its optimal performance in terms of metabolic activity and kinetics of cell cultures, economic costs, and its flexible operation regarding maintenance of cultures by controlling operational conditions, such as temperature, pH, aseptic, mixing, aeration, and scalability. Table 4 shows some characteristics, advantages, and disadvantages of these types of bioreactors. 

#### Engineering Aspects in the Plant Cell Suspension Culture

Engineers designing or optimizing bioreactor technologies must both consider the effect of operating conditions on the complex interaction between transport phenomena, thermodynamics, growth kinetics, metabolic activity, and maintenance of plant cell cultures and, based on it, propose methodologies to transfer information observed in flask cultures to larger bioreactor scales. Some operational conditions are critical because they can cause a decrease in biomass, a low PDSM production, or a loss of cell viability. Table 5 shows some CSC that have been successfully scaled from flask cultures to large-scale bioreactors.

The scaling up of CSC carried out in a flask culture demands the use of bioreactor engineering to characterize the impact of operating conditions on growth kinetics, cell deactivation, and transport phenomena and, hence, on the metabolic activity and production rates of PDSM. To this end, in what follows, main aspects to be considered during the scaling up of CSC, from the screening of plant cells to the industrial-scale bioreactor design, are mentioned and analyzed.

The screening of a set of plant cells is considered as the first stage during the scaling up of CSC [42,94]. Screening takes place in shake flasks. In these laboratory bioreactors, hydrodynamic and transport phenomena negatively impact on the growth kinetics, cell viability, metabolic activity, and production rates of PDSM. For instance, in these bioreactors, the production of PDSM involves two-phase systems (liquid culture phase and cell culture phase) neglecting the effect of operating conditions, including the impact of the oxygen concentration, on the microscopic and macroscopic performance of the shake flask. In this context, apparent results regarding cell growth kinetics, cell viability rates, and production rates of PDSM are observed. In these conditions, promising plant cells are identified and selected to be evaluated in larger bioreactor configurations, such as those presented in Table 4.

The second step accounts for characterization of cell growth kinetics, cell viability rates, metabolic activity, and production rates of PDSM under controlled operating conditions in bench-scale bioreactors with similar configurations to those systems to be implemented at the commercial scale, i.e., bench-scale bioreactors accounting for three phases (liquid–gas–cells) (see Table 4). Thus, during the analysis of bench-scale systems, the coupling of experimentation with mathematical modeling is essential for stating the basis for the scaling up of CSC [130,131,132]. Herein, cell growth kinetics and production rates of PDSM are the main response variables to maximize during CSC. It is worth mentioning that their experimental and theoretical characterization makes possible the connection between the microscopic world of the metabolic cell activity and the macroscopic world of the bioreactor performance and, hence, the downstream processing. Besides, the experimental characterization of these cell mechanisms and their analysis using mathematical models lead to the construction of the engineering tool for the scaling up and optimization of the bioreactor configuration, allowing a better understanding of CSC during the production of PDSM. In particular, the use of bench-scale bioreactors allows for identifying and controlling those operating conditions where transport phenomena favor the kinetics of the CSC.

Based on the kinetics, since in CSC it is not possible to develop intrinsic kinetic models, there are two types of models that can be developed in bench-scale bioreactors: extrinsic ones, where transport phenomena are explicitly included during the modeling of the bioreactor; and apparent ones, where transport phenomena resistances impact during the experimentation but they are not considered during the modeling of the bench-scale bioreactors [131,133,134,135,136,137]. Thus, to determine extrinsic kinetic models, it is recommended to carry out a regime analysis to identify and model those transport phenomena limiting the production of PDSM. Experiments make possible the development of the corresponding model, relating kinetics with macroscopic variables, namely the concentration of substrates and PDSM, cell growth, and cell viability involved during the operation of the bench-scale bioreactor. The kinetic model depends on the quality of the experimental data and it is only reliable for the range of operational conditions utilized during its development. When the kinetic model is based on metabolic steps of the reaction, the mathematical complexity increases but leads to a better physical representation of the CSC during the production of PDSM. Besides, the loss of cell viability caused by operational aspects, i.e., a toxic compound, cell shear stress, or cell sintering, is modeled by empirical expressions whose parameters involve physical meaning [138], such as the generalized power law equation (GPLE) [139,140,141]. Finally, the Monod model offers an adequate explanation for the reaction rates of growing cells, but it has no mechanistic basis [142,143]. Moreover, the Monod model is only applicable when cells are in a metabolic equilibrium, namely when the composition of the macromolecules in the cell remains in a pseudo-steady state during the CSC. Table 6 presents some kinetic models to describe cell growth rate. It is worth mentioning that, in transient experiments, when the concentration of a substrate or PDSM is brusquely modified, Monod kinetics are not suitable and the kinetic model must account for the cell metabolism [138,144]. There are, in the literature, several models that have no mechanistic grounds but account for some biological features of the cell growth [138,145]. These models offer an acceptable description of the cell growth and metabolic activity due to fluctuation in the concentration of substrates and products. In these models, cell mass is divided into compartments, and the rate of formation of each compartment has different stoichiometry and kinetics.

In bench-scale bioreactors, it is experimentally complicated to minimize transport resistances [139,140,141,146]. In the fluid bulk, concentration, temperature, or radiative gradients can be present. Hydrodynamics impact on mass and heat transfer mechanisms from the gas phase to the liquid phase and from the liquid phase to the cell phase. Moreover, cell growth can impact on mass and heat transfer mechanisms. Although complicated, a proper kinetic analysis must account for the effect of fluid dynamics on transport phenomena and, hence, on cell growth, cell viability, and metabolic activity.

During the screening at the laboratory bioreactors or during the operation of the bench-scale bioreactor, the response surface methodology (RSM) is a potential tool to guide experimental designs. RSM leads to the following advantages [147,148,149,150,151]:

(1) It defines an establishment of the relationship between responses (yield, cell viability, oxygen concentration, etc.) and control operating conditions (temperature, pressure, initial concentration, power input, agitation rate, etc.).
(2) It predicts the effect of control operating condition on responses.
(3) It gives inferences on the significance of the operating conditions on the performance of the reactor.
(4) It allows the determination of the operating window where the bioreactor meets its best performance.

On the above end, RSM couples experimental designs, and mathematical and statistical methods [152,153]. Firstly, an experimental design is proposed; the evaluation of this experimental design constitutes the so-called response surface design (RSD). The suitability of the RSD depends on its orthogonally, ratability, and uniform precision [153]. Secondly, the empirical model is then developed; it is approximated by a polynomial equation that accounts for elements that consist of powers and cross-product powers, constant coefficients referred to as parameters, and a random experimental error. Albeit empirical, first-degree and second-degree polynomial equations are usually used to fit observations and carry out the optimization. To this end, every model and its reliability depends on the RSD, i.e., first-order designs are used to fit observations with the first-degree models, and observations out of second-order designs are fitted with second-degree models [152,153,154]. The most common first-order designs are 2k factorial, Plackett–Burman, and simplex designs, while the most common second-order designs are 3k factorial, central composite, and the Box–Behnken designs. Note that the choice of a proper RSD is essential since the quality of prediction, as measured by the size of the prediction variance, depends on it; thus, the lower the variance, the better the fit of the responses. On this basis, a single RSD is not able to satisfy all criteria, but it is considered as robust if it meets the assumptions related to the model and the error distribution [152,153]. Finally, the assessing of the results uses both statistical tests, i.e., F-value, t-value, and confidence interval, and graphical tests, i.e., variance dispersion graphs, fraction of design space plots, and quantile plots. Graphical methods [149,150] based on quantile dispersions have also been used to compare experimental designs for estimating variance components in an analysis of variance (ANOVA) situation. RSM can lead to the identification of the operational window where CSC presents its higher yields to PDSM, which, in turn, will be essential in the conceptual design and scaling up of the bioreactor configuration.

Because of the advent of computation in the last years, the bioreactor design not only depends on empirical, but also deterministic approaches, which allows the proper determination of hydraulics, fluid dynamics, mass transport, heat transfer, radiative transfer, and kinetics from different bioreactor configurations at various scales. This information is transferred to design and scale up the industrial bioreactor. The design of this reactor strongly depends on the development of a model coupling kinetics and transport phenomena at both the cell and bioreactor level, including the fluid and the gas phase. This is, however, a complex task, since it needs experiments and mathematical solutions that are not trivial. It is worth stressing that, during the construction of this model, fluid dynamics are yet the bottleneck during the scaling up of a bioreactor configuration because of their impact on transport phenomena, kinetics, and, hence, on the global production of PDSM.

Based on the above, a model accounting for kinetic, deactivation, and all transport mechanisms should be developed from the laboratory to the bench scale. This model should be constructed following a framework based on computational fluid dynamics (CFD). The model needs to be validated at the bench scale before using it to design the industrial bioreactor. The preliminary dimensions of the reactor need to be obtained from the utilization of the practical know-how reported in the literature or experimental and modeling results obtained at the bench scale. It will make the scaling up process more efficient and reliable. Developing a model for the use of CFD allows the consideration of fluid dynamics along with its effect on transport phenomena, which leads to obtaining operating conditions where mixing, hydrodynamics, and transport phenomena are improved without affecting the operating cost of the process. A criterion when designing the industrial-scale bioreactor is to achieve a compromise between operating expenses and yield of the PDSM. At the end of the scaling-up process, the experimentation and investment cost as that compared using an empirical or heuristic approach will be significantly minimized.

In addition to the aforementioned, the scaling up of CSC becomes more challenging when observing how operating conditions impact on the production of PDSM. Operating conditions influence in different scenarios and magnitudes the performance of cell cultures during the production of PDSM, from the supply of nutrients (oxygen, light, ionic strength, pH) to the implementation of mechanical and pneumatic work to keep the process operating in optimal conditions. In further sections, a discussion about the main operating variables in bioreactors and their effect on the performance of cell culture will be provided.

### 3.5. Effect of Operating Variables on the Bioreactor Performance

#### 3.5.1. Temperature

It is a key variable that must be kept under control because its increment impacts on kinetics, inducing the premature senescence of the culture, and produces the loss of viability, such that it reduces the yield of PDSM. On the other hand, temperature may also act as an abiotic elicitor. Temperature in bioreactors has varied between 23 and 30 °C [155,156], although most of the works have fixed it at 25 °C [122,123]. Most temperature control systems consist of the use of temperature probes and jackets or coils used as a heat transfer system to activate metabolic reactions or cool the bioreactor because of the heat generated by exothermic metabolic reactions. Heating or cooling systems also used in bioreactors are electric heaters or steam streams for the former, and cooling water or refrigerants in cooling towers for the latter [115]. Species such as *Catharanthus roseus* and *Lavanda vera* exhibited the best kinetic cell performance at 30 °C, leading to a constant production rate of PDSM when CSC was carried out in stirred tank bioreactors [157,158].

#### 3.5.2. Light

Cell irradiation by use of visible light is one of the most important elements when designing CSC bioreactors, since photons affect the growth and morphogenesis of cell cultures both in in vivo and in vitro conditions. Plant cell cultures vary their physiological response to the light exposure because of photolytic reactions, significantly influencing the synthesis and production of secondary metabolites, such that yields are a function of the type of species, growth stage, type of light, and time of exposure to light. In plant cell cultures, the use of 12/16 h light/dark photoperiod is the most frequent condition, although total darkness and continuous light exposure have also been reported [52,159,160]. Some examples recently reported in the literature about the effect of light exposure in CSC are for *Vitis vinifera* [159], *Theobroma cacao* [161], *Clinacanthus nutans* (Burm. f.) Lindau [162], *Catharanthus roseus* (L.) G Don. [163], and *Artemisia absinthium* [164], where higher production of PDSM were obtained under light exposure. On the other hand, species such as *Plumbago europaea* L. [160] showed 1.7 times the production of plumbagina, the main bioactive compound, when CSC were cultured in darkness; besides, *Ajuga bracteosa* exhibited a higher concentration of PDSM and antioxidant activity when its CSC was subjected to darkness and methyl jasmonate as elicitor [165], and *Ruta graveolens* with a 3.14-fold increase in total flavonoid content [166].

#### 3.5.3. pH

pH is one of the factors influencing the growth and production rates of PDSM; therefore, it is a critical operating condition in small- and large-scale plant cell culture. It provides the proper balance of acidity/alkalinity to the culture to avoid cell breakage. The initial pH in plant cell cultures usually ranges between 5.5 and 6.0; abrupt changes during the culture can cause variations or loss of the nutrient uptake. One of the most relevant challenges during the operation of the bioreactor is the successful implementation of an efficient pH control system. The bioreactor behavior depends on the change in pH, modifying the cell growth and the production of PDSM and being widely dependent of the species related to the plant [13,156,167,168].

#### 3.5.4. Mixing

In cell cultures, the production of biomass and PDSM is highly dependent on the conversion of substrates into products, this conversion and reaction rates are mediated and controlled by transport mechanisms toward and from the place where the conversion occurs. In every bioreactor, fluid dynamics impact how substrate and inoculum are transported to the liquid bulk and how PDSM are removed. In batch operations, fluid dynamics occasioned by mixing prevents local exhaustion of substrates such as oxygen [169]. Mixing and fluid dynamics are a function of the bioreactor configuration and impact the performance of CSC, such that, when properly controlled, they minimize interfacial mass and heat transport resistances, decreasing temperature and concentration gradients in the bioreactor bulk and, hence, having homogeneous distribution of the components or conditions in the culture medium [170].

The characteristic time related to mixing in bioreactors influences heat production, oxygen mass transfer, and C-substrate consumption in the cultures [24,169]. For instance, the characteristic time related to heat production (mainly due to metabolic heat) is defined as the time necessary to heat up the content in the vessel by 1 °C, while the mixing characteristic time is usually considered as the one for smoothing out temperature gradients occasioned by the metabolic reactions. In most bioreactor configurations, mixing times range from 10 to 100 s, such that heat will not be accumulated.

The characteristic time for mass transport is defined as the characteristic time required for the decrease in oxygen concentration once the gas flow rate is discontinued, this time is not related to a critical time for the micro-organism. Depending on the bioreactor configuration, the capacity of oxygen transfer rate (OTR) will vary [170]. Despite STR exhibiting better OTR, it has some restrictions related to the dispersion of gas through the vessel, because the higher transfer occurred near the sparger, reducing the concentration of dissolved oxygen in the aqueous medium. In this sense, the respective characteristic time for oxygen depletion in STR is considered as the critical time when it is assumed that all the oxygen is transferred close to the impeller. On the other hand, bubble column bioreactors are not excluded for exhibiting oxygen depletion; in this configuration, the oxygen is transferred at all the positions in the column, and even though the oxygen transfer is mainly observed near the sparger, it is greater than that for STR. Thus, the characteristic time for depletion is based on the concentration of oxygen dissolved outside the region of the sparger [170,171,172].

The last characteristic time associated to the mixing process in bioreactors is related to the relationship between cell growth rate and bulk substrate concentration. Since the Monod model describes adequately this phenomenon, this equation is used to determine the characteristic time for substrate consumption. This characteristic time will be larger than the one for mixing time and fluid dynamics calculated for the bioreactor. Thus, cell growth rate and consumption substrate rate strongly depend on the transport phenomena resistances and, hence, characteristic times involved in every bioreactor configuration.

##### Considerations of Cell Culture Properties on the Mixing Process

Plant CSC is effectively set up in small-scale bioreactors, exhibiting excellent production of secondary metabolites, but, when it comes to working with larger scales, the situation is not that simple. Cell cultures tend to form flocs and agglomerates when the daughter cells are not completely separated from the stem ones after cell division; these agglomerate systems are constituted from several hundreds of cells, ranging in particle sizes around 0.5 cm diameter, depending on the cell line, culture conditions, and growth stage [16,111,173]. These agglomerated structures lead to the formation of heterogeneous populations creating microenvironments (larger intra-agglomerate transport resistances) that limit the substrate and oxygen transfer rates, causing low rates of growth and PDSM production. Some studies have demonstrated that the production of specialized high value-added PDSM depends on the formation of aggregates and their size [174,175,176,177]. For example, growth characteristics and qualitative composition of PDSM in *Phlojodicarpus sibiricus* cell cultures were directly correlated with the level of cell aggregation, being more favorable in aggregates of 10–30 cells than in aggregates of >50 cells [174]. Small aggregates (~400 µm) within *Taxus* suspension cultures produced four times more paclitaxel than larger aggregates (~1100 µm) [178].

However, despite intra-aggregate diffusion limitations, it has been reported that the formation of large aggregates may favor the production of PDSM, as is the case with cell suspension cultures of *Psoralea corylifolia*, where an aggregate size of 1200–2000 µm favored the production of phytoestrogens compared to sizes of 800–1200 µm [179]. Moreover, the formation of cell aggregates can also be favored by the secretion of extracellular polysaccharides (ECP), contributing to greater cell adhesion. Therefore, aggregation patterns coupled with high biomass concentrations and ECP secretions result in culture mediums with non-Newtonian characteristics [180].

One of the most logical and simplest ways to overcome these concerns could be related to the increase in the stirring speed, because it will break the flocs and cell agglomerates, facilitating the substrate and oxygen uptake and, therefore, increasing the growth rate and conversion to desired products [174,181]. Nevertheless, plant cells, as with other micro-organisms, are sensitive to shearing, and surpassing their resistance umbral to hydrodynamic stress may induce their cell wall breakage, causing the loss of valuable products [124,180,182]. Besides, plant cell cultures are characterized by being viscous and highly dense suspensions that behave as non-Newtonian fluids, which also contributes to restricting the flow regime and the heat and mass transfer mechanisms, leading to zones with gradients of concentration and temperature, and, therefore, to dead zones of mixing in the bioreactor [42,173]. For this reason, in CSC the mixing is often evaluated in terms of its impact on the biological performance (growth rate and productivity) of the bioreactor technology.

A clear example that the aggregate size is an important parameter for the production of high added-value PDSM is the production of Paclitaxel by *Taxus chinensis* cultures. The authors of [176,181] showed that mechanical shear helps disintegration, favoring production by having small aggregates (194 µm) compared to the control, in which large aggregates (600 µm) were obtained that directly affected production.

#### 3.5.5. Aeration

Aeration, as well as mixing, is one of the most important operating variables, both acting synergistically in bioreactors by maintaining aerobic conditions [183], helping to desorb volatile products, eliminating the metabolic heat, contributing to the synthesis of PDSM, and having a beneficial effect on power consumption [10]. In most of bioreactor configurations, gas stream is split by highly porous spargers in the form of bulb diffusers, sintered filters, or perforated plates, where gas bubbles are generated on the bottom of the vessel and rise through the culture medium, producing pneumatic mixing. The mass transfer will depend on the type of sparger and gas flow rate. Despite physiological differences between microbial and plant cell cultures, the use of excessive aeration conditions might cause foam generation due to the presence of extracellular proteins [184].

CSC in plants generally exhibits a doubling time of about 2–5 days, longer than that required by bacteria cells (0.5–1 h) [42,185]. For this reason, slower growth rates in plant cells lead to low oxygen demand, with a direct relationship to the cell concentration. High cell densities in the bioreactors may not be as desirable because it can cause limitations and inadequate concentrations of dissolved oxygen. The oxygen uptake rate (OUR) in CSC is commonly used for monitoring the physiology and oxygen demand by plant cells; this parameter is dependent on the cell culture line, culture conditions, and the growth rate [170]. Typical OUR values for plant CSC range from 5 to 10 mmol O_2_/L h), a lower requirement when compared to that for microbial cells (10–90 mmol O_2_/L h). Another important parameter considered for the establishment of aeration conditions is the oxygen transfer rate (OTR); it must be high enough to provide the required oxygen concentration to meet the respiratory demands of the cells (OUR), favoring the growth and production of the desired compounds, but not too high that it can hinder them. To overcome these difficulties, the concentration of dissolved oxygen must be kept above the critical level of cell oxygen consumption, which has been reported to be 15–20% oxygen saturation content in pure water (1.3 to 1.6 g/m^3^) [11,77]. Dissolved oxygen concentration is not a variable that can be used for scaling criteria, and instead the volumetric mass transfer coefficient (kLa) and the air flow rate are used (vvm). kLa is a function of both agitation and aeration and is affected by various factors, such as geometric and operational characteristics of the reactor (stirring speed, aeration rate, fluid hydrodynamics, media composition, cell type, morphology, and concentration), which must, therefore, be analyzed when designing the bioreactor [186]. In agreement to the literature [24], to achieve an OUR around 5 to 10 mmol O_2_/L h) in plant CSC, a typical kLa value between 10 and 50 h^−1^ is required. In Table 7, some of the operating conditions used in bioreactors and their effect on the CSC are described.

Other variables related to the aeration process in bioreactors are the superficial velocity of the gas, which permits the calculation and inference of the air bubbles’ behavior in the bioreactor, and the observation of their coalescence in the medium. The superficial velocity is controlled by the aeration rate [119,187] showed that plant cell cultures, when subjected to hydrodynamic stresses, can change their color and increase the PDSM production as a defense mode. Nevertheless, as mentioned above, aeration also has an effect on the mixing and mass transfer, so low aeration conditions will lead to poorly homogeneous conditions and limitations on cell growth due to the presence of sedimentation, or the development of microenvironments due to concentrations gradients. On the other hand, the increase in aeration could overcome these disadvantages, but also, the increase in shearing occurrs, making it necessary to establish the fragility of the cells to define the intensities of aeration that maintain an adequate level of homogeneity, without affecting the cell growth nor the production of PDSM.

**Table 7:** Conditions used in bioreactors and their effect on the SCC.

Species	Compounds	Bioreactor	Operating Conditions	Operation Variables	Effect of the Operating Variable	Ref.
*B. cordata*	Phenolics (phenylethanoid glycoside and flavonoid contents)	STR of 2 L (ring diffuser) and 3 L (sintered diffuser), Rushton impeller	26 ± 2 °C, photoperiod of 16 h light (50 µmol/m^2^ s)/8 h darkness Fg: 0.1 vvm	Stirring speeds (120 and 400 rpm)	In both bioreactors, a higher shear stress was observed at rates of 400 rpm, affecting the growth phases and parameters, resulting in the decrease in PDSM.	[124]
*R. cordifolia*	Anthraquinones	STR of 8 L	25 ± 0.1 °C, gamma-irradiated cell cultures, the agitation speed of the impeller was 60 rpm, working volume 5 L	Impeller type (helical ribbon, Rushton turbine)	Helical ribbon provided a homogeneous mix and lower shear stress compared to Rushton turbine.	[66]
*R. tinctorum*	Antraquinones	Baffled flask	25 ± 2 °C, the cultures were grown in presence or in absence of light with a 16 h photoperiod using cool white fluorescent tubes at a light intensity of approximately 90 mol/m^2^ s	Stirring speeds (100, 360 rpm)	The speed at 360 rpm had a negative effect on cell growth; however, it favored the production of PDSM	[188]
*R. tinctorum*	Antraquinones	STR of 1.5 L, turbine impeller	25 ± 2 °C, working volume of 1.0 L Fg: 1 vvm	Shear stress (450 rpm)	The speed of agitation affected cell viability; however, it favored the production of PDSM.	[125]
*Arnebia* sp.	Shikonin	Air-lif of 2 L	25 ± 2 °C, the dissolved oxygen (2 L/min)	Bioreactor-type	No significant differences were obtained in the growth and production of PDSM in both bioreactors.	[126]
		STR of 2 L, six-blade turbine impellers	25 ± 2 °C, 100 rpm, dissolved oxygen (2 L/min)			
*V. officinalis*	Phenylpropanoid glycosides (Verbascoside) (Isoverbascoside)	STR	23 ± 1 °C, photoperiod, 33 rpm and continuous Fg: 0.5 vvm	Bioreactor-type	The production of PDSM was significantly higher in the STR bioreactor	[92]
	Phenolic acids (Ferulic and Rosmarinic acid)	Balloon bioreactor (BB)				
*T. minus*	Berberine	STR of 2 L, Rushton turbine	25 °C in the dark, working volume (1.75 L) Fg: 0.1 vvm	Stirrer speeds of 100–900 rpm	The 250-rpm speed favored cell growth and PDSM production	[77]
			25 °C in the dark, working volume (1.75 L) and 250 rpm Fg: 0.1 vvm,	Dissolved oxygen fluctuations (25, 35 and 50%)	Fluctuations in dissolved oxygen tension affected berberine accumulation in the *T. minus* cultures depending on the average oxygen level achieved. Reductions in berberine production were observed not only as the average dissolved oxygen tension declined below 35% air saturation	
*D. deltoidea*	Steroid glycosides	BC of 20 and 630 L	26 ± 0.5 °C in darkness, working volume of 15 L and 550 L, semi-continuous regime. Fg: 0.1 to 1.0 vvm depending on the growth phase of cell culture, OD was maintained at 10–40% of saturation volume	Bioreactor volume	No significant effect of bioreactor volume was obtained on cell growth and PDSM production.	[182]

PDSM: plant-derived secondary metabolites; STR: stirred tank reactor; Fg: aeration flow rate; vvm: gas volumetric flow rate per unit volume of culture medium.

## 4. Conclusions

To conclude, plants are a rich source of bioactive compounds of pharmacological interest, known as PDSM; due to their low production in nature, obtaining them leads to overexploitation and extinction of the species of interest. As an alternative ecological solution, plant cell culture, particularly CSC, stands out as one of the most efficient and promising technologies for producing PDSM in bioreactors. The selection and design of the bioreactor for the production of PDSM out of CSC is a complex task, which depends on two factors: on the one hand, the properties of the cells that vary according to the species under study (shear stress, aggregate formation, and rheology) and, on the other hand, transport phenomena related to the bioreactor configuration. The operating conditions in the bioreactor impact cell performance, such that the mixing and aeration are factors influencing fluid dynamics and, hence, mass transfer and heat transfer at both inter- and intracell levels. Thus, the optimization of bioreactors by elucidating the effect of the operating condition on the cell properties is essential for obtaining larger yields of PDSM. Despite the arduous research in this field, few PDSMs are commercialized at an industrial level. To this end, more studies focused on correlating the operating variables with kinetics and transport phenomena are needed to understand the behavior of plant cells, providing more bases for optimal growth and maximum production of PDSM in bioreactors.

This review provided updated information that helps the reader to understand the behavior of plant cells growing in suspension, identifying the key parameters to relate PDSM productivity with the optimization of operating variables in bioreactors, which will help future research in the scaling of PDSM with high added value, resulting in the development of new successful biotechnological processes.

## Figures and Tables

**Table 2:** PDSM from medicinal plants successfully produced in the in vitro plant cell culture, bioactivities, and yield comparison.

Compound	Plant Species	Biological Activity/ Pharmaceutical Use	Extraction Yield		Type of Culture	Ref.
			Mother Plant	In Vitro Cell Culture		
Shikonin	*Lithospermum erythrorhizon* *Alkanna tinctoria* Tausch	Anticancer, antibacterial, anti-inflammatory, hepatic steatosis attenuator, antitumor, and antioxidants	10−20 mg/g	150−200 mg/g	CSC	[58,59,60,61,62]
	*Echium plantagineum* L.			36.25 mg/L	HRC	[63]
Anthraquinones	*Morinda citrifolia* *Rubia cordifolia* *Senna obtusifolia*	Antimicrobial, antifungal, hypotensive, analgesic, antimalarial, gastroprotective, antioxidant, hepatoprotective and antileukemic, and mutagenic functions	3 mg/g	100–200 mg/g	HRC CCC CSC	[64,65,66,67]
Rosmarinic acid	*Ocimum basilicum*	Antioxidant, anti-inflammatory, antiviral activities	8.78–9.4 mg/g	12.32–21.28 mg/g	CSC	[68,69]
	*Origanum vulgare*		23.53 mg/g	31.25 mg/g	CSC	[70,71]
	*Satureja khuzistanica*		12 mg/g	38 mg/g	CSC	[72,73]
	*Coleus blumei*		30 mg/g	270 mg/g	CSC	[32]
	*Salvia officinalis*		30 mg/g	360 mg/g	CSC	
Berberine	*Thalictrum minus*	Effects antitumor, anticancer, lower blood lipid, lower blood glucose, anti-osteoporosis, anti-osteoarthritis, antibiotic, and anti-inflammatory	0.1 mg/g	0.8 mg/mL	CSC	[74,75,76,77,78,79]
	*Coptis japonica*		20–40 mg/g	132 mg/g	CSC	
	*Coscinium fenestratum*		1 mg/g	178 mg/g	CCC	
Ginsenosides	*Panax ginseng*	Antitumor, immunological, anti-inflammation, anticancer, antidiabetic, and cardiovascular-protective	0.015–8 mg/g	36.4–80 mg/g	HRC	[57,80,81]
				3.4–28.9 mg/g	CSC	
				15.1–105.6 mg/g	ARC	
	*Panax japonicus*			20–50 mg/g	CSC	
	*Panax notoginseng*			60 mg/g	CCC	
				71.94 mg/g	ARC	
				40 mg/g	CSC	
Diosgenin	*Dioscorea deltoidea*	Anticancer, antidiabetic, anticoagulant, antithrombosis, anti-inflammatory, antiviral, anti-ageing	0.4−3 mg/g	72 mg/g	CSC	[82]
				3.5–16 mg/g	CCC	
	*Dioscorea bulbifera*			12 mg/g	CCC	
	*Helicteres isora* L.		1–5 mg/g	8.64 mg/L	CSC	[83]
				23 mg/g	CCC	[84]
Ajmalicine	*Catharanthus roseus*	Antihypertensive, obstructive circulatory diseases treatment	3 mg/g	63 mg/L	CCC	[85]
				10 mg/g	CSC	[86,87]
				34 mg/L	HRC	
Paclitaxel	*Taxus chinensis*	Anticancer	0.02 mg/g	1.5 mg/g	CSC	[88]
Podophyllotoxin	*Linum narbonense*	Vigorous antimitotic and antiviral activities and anticancer	0.5 mg/g	1.57 mg/g	CCC	[89]
	*Juniperus chinensi*		0.025 mg/g	189.91 mg/g	CSC	
	*Linum flavum*		1.6 mg/g	2 mg/g	CSC	
Artemisinin	*Artemisia annua* L.	Treat multi-drug-resistant strains of falciparum malaria	1–15 mg/g	9.33–110.2 mg/L	CSC	[90,91]
Phenolic Acids	*Verbena officinalis*	Antimicrobial, secretolytic, expectorant, and diuretic agent	136.59 mg/g	126.55 mg/g	CCC	[92]
(rosmarinic, chlorogenic, and ferulic acid)				189.91 mg/g	CSC	
Resveratrol	*Vitis vinifera* L.	Reduced coronary heart disease mortality rates and atherosclerosis, inhibiting low-density lipoprotein oxidation, and carcinogenesis	NR	277.89 µg/g	CSC	[7]

CSC means cell suspension culture; HRC means hairy root culture; CCC means callus cell culture, ARC adventitious root culture; NR means not reported.

**Table 3:** Plant-derived products manufactured from plant CSC which have entered into the pharmaceutical industry. The list of products makes no claim to be complete.

Product	Species	Pharmaceutical Use	Manufacturer, Tradename, and Scale of Production	Type of Culture	Reference
Rosmarinic acid	*Coleus blumei*	Anti-inflammatory	ANattermann & Cie. Gmbh, www.sanofi.de (accessed on 30 October 2021)	CSC	[103]
Echinacea polysaccharides	*Echinacea purpurea*	Immunostimulant, anti-inflammatory	Diversa, 75,000 L bioreactor	CSC	[100,104]
Berberines	*Thalictrum minun*	Anticancer; antibiotic; anti-inflammatory	Mitsui Chemicals, Inc., (75,000 Lbr)	CSC	[105]
	*Coptis japonica*		https://www.mitsuichemicals.com/ (accessed on 30 October 2021)	CSC	
Podophyllotoxin	*Podophyllum* spp.	Anticancer	Nippon Oil Company, Ltd.	CSC	[106]
			https://www.freepatentsonline.com/5336605.html (accessed on 30 October 2021)	OC	[107]
Docetaxel	*Taxus baccata*	Ovarian cancer treatment	Phyton Biotech, Inc., Taxotere (150 kg/year)	CSC	[108,109]
			https://phytonbiotech.com/ (accessed on 30 October 2021)		
Paclitaxel	*Taxus* spp.	Anticancer: FDA approved for the treatment of ovarian, breast, and lung cancers	Phyton Biotech, Inc., Taxol ^®^ (1000 kg/year)	CSC	[110]
			https://phytonbiotech.com/ (accessed on 30 October 2021)		
			Samyang Genex Corporation., Genexol (32,000 Lbr) https://samyangbiopharm.com/eng/ProductIntroduce/injection01 (accessed on 30 October 2021)	CSC	[111]
					[112]
Scopolamine	*Duboisia* spp.	Anticholinergic; antimuscarinic; motion sickness, nausea, and intestinal cramping	Sumitomo Chemical Co., Ltd., Tokyo, Japan (50–20,000 Lbr) https://www.sumitomo-chem.co.jp/pharma-chem/ (accessed on 30 October 2021)	HRC	[113,114]
Shikonin	*Lithospermum erythrorhizon*	Anti-HIV, antitumor, anti-inflammatory	Xi’an NEO Biotech, Shikonin 95%	CSC	[100]
			http://www.extractneo.com/about (accessed on 30 October 2021)		

CSC: cell suspension culture; HRC: hairy root culture; OC: organ culture.

**Table 4:** Comparison of bioreactor configurations commonly used for plant cell culture.

Bioreactor Configuration	Schematic Diagram *	Description	Advantages	Disadvantages	Ref.
Bubble column (BC)	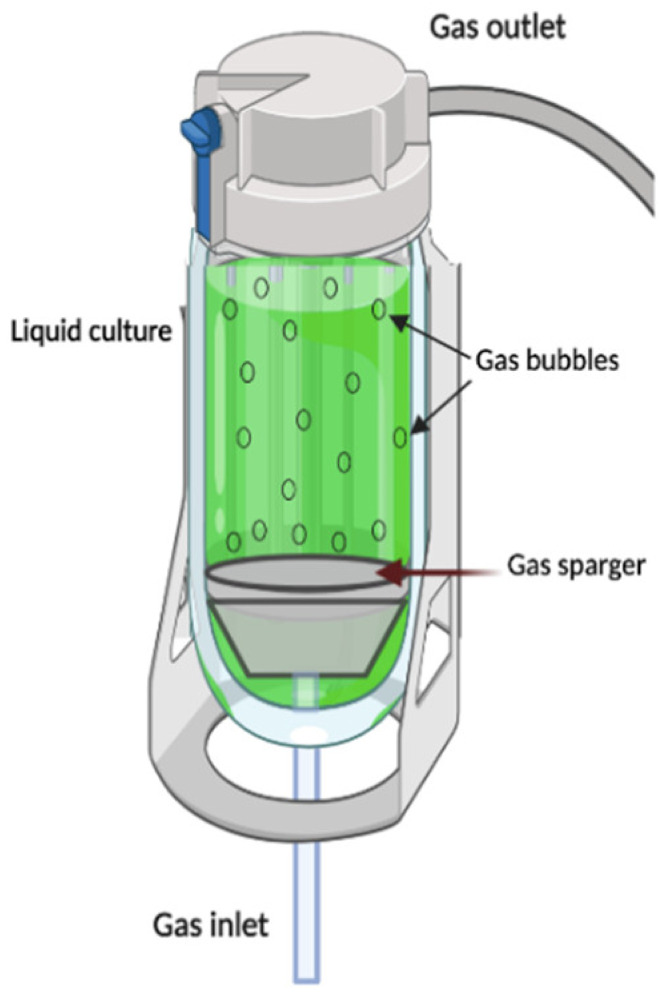	It is classified in the pneumatic-type bioreactor. They are constructed in cylindric columns where gas injection represents the only energy entrance to the system. BC bioreactors operate under constant bubbling where gas flows from the bottom to the top through nozzles, perforated plates, or spray rings, allowing not only the aeration process, but also helping the mixing and circulation of the fluid, without the need to install mechanical accessories.	Simple structure as no mechanical force is required to shake. Easier maintenance and reduces the risk of contamination due to the lack of mobile parts. Reduced effect of the shear stress.	High foam formation under high gas flow rates. Poor oxygen transfer capabilities. Poor fluid mixing in highly viscous fluids. High levels of foaming under high-aeration conditions	[24,94,116]
Airlift (ALB)	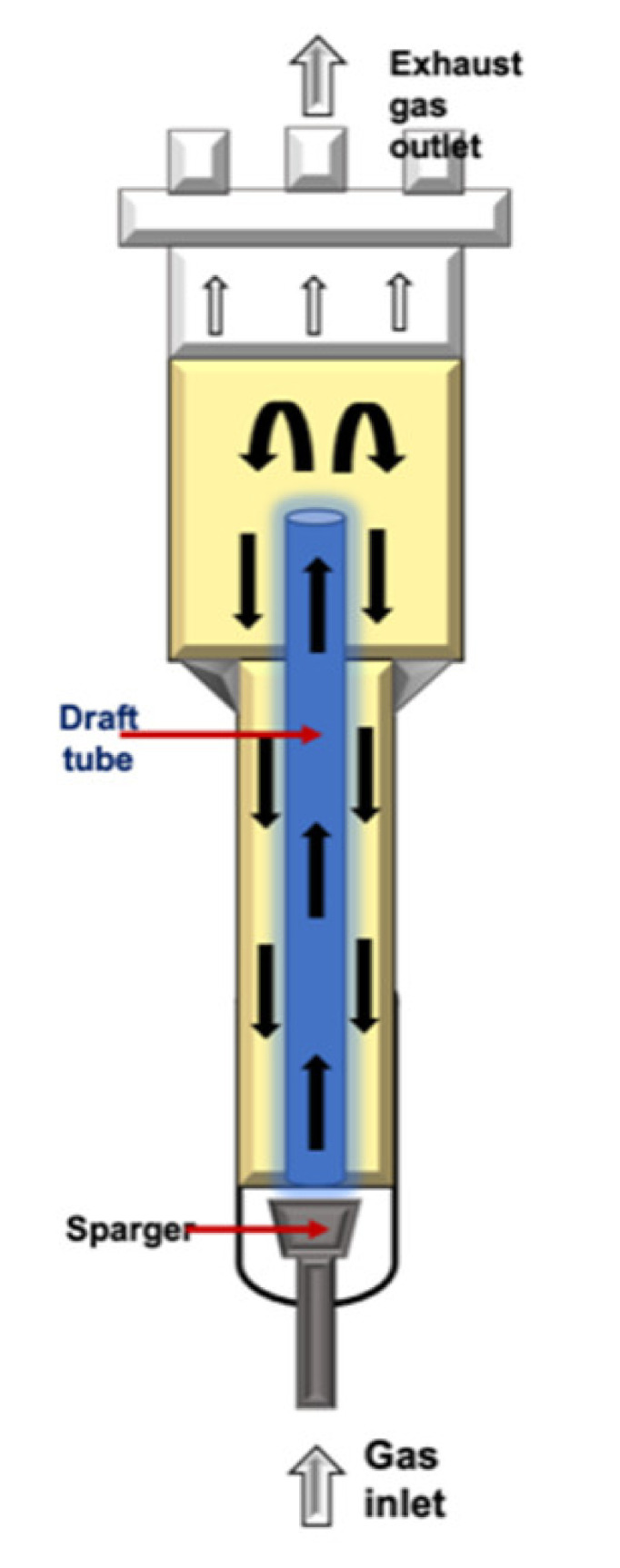	It is classified in the pneumatic-type bioreactor. This configuration is considered reasonably like STR, excepting for the impeller. They are tower reactors where fluid broth is mixed with a gas stream, which is compressed and injected at the bottom of the discharge pipe. The gas–fluid mix allows the creation ofdifferences in density and upward displacement. It is more suitable for hairy root and somatic embryo cultures.	Easy maintenance and reduces the risk of contamination due to the absence of mobile parts. Reduced effect of the shear stress. Higher oxygen transfer than that in BC. The energy required is provided by the compressed gas.	High levels of foam formation under high gas flow rates. Poor fluid mixing in highly viscous fluids. Relatively poor oxygen transfer capabilities.	[24,117,118,119]
Stirred tank bioreactor (STB)	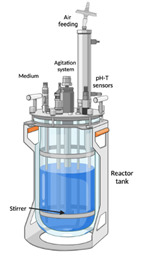	It is grouped in the mechanically agitated bioreactor. This bioreactor consists in a mixer (turbine or propeller) installed within the tank reactor and may be equipped with gassing inlet stream. It can operate in batch, semi-continuous, or continuous mode [117,120].	Efficient fluid mixing systems. High oxygen mass transfer capability. Convenient for high-viscous fluids. Comply with Good Manufacturing Practices. Easy scale-up. Highly adaptable to production scale and products. Impeller alternative.	High energy cost owing to mechanical agitation. Contamination risk with mechanical seal. Some cells and metabolites are susceptible to shearing generated by the impeller and bursting gas bubbles. Depending on the operation mode, this configuration can represent high costs of maintenance, cleaning, and startup.	[94,117,120,121]

* Schemes were created with BioRender.com.

**Table 5:** Comparison of operating conditions used for SCC in flask and bioreactor to produce PDSM. The list of examples makes no claim to be complete.

Species	Compounds	Operation Variables Evaluated		Biomass Production		PDSM Production	Ref.
		In Shake Flask	In Bioreactor	In Shake Flask	In Bioreactor		
*Scrophularia striata*	Phenylethanoid glycosides	50 mL SCC in 100 mL flask 110 rpm 25 °C	5.0 L SCC in STR 10 L Fg: 0.5–1.0 L/min 110−170 rpm 25 ± 1 °C Darkness	14.16 g/L	15.64 g/L	The acteoside content in CSC in the bioreactor was about threefold higher than that in the shake flask	[122]
*Buddleja cordata*	Verbascoside, linarin and hydroxycinnamic acids	50 mL SCC in 250 mL flasks 110 rpm 26 ± 2 °C	STR 2 L Fg: 1 vvm (ring diffuser Rushton impeller 400 rpm 26 ± 2 °C 16/8 h light to dark photoperiod	11.8 g/L	13.62 g/L	The content of phenolics was twofold higher in STR.	[123,124]
*Rubia* *tinctorum*	Anthraquinone	25 mL SCC in 250 mL flasks 100 rpm 25 ± 2 °C 16/8 h photoperiod (140 µmol m^−2^ s^−1^)	1.0 L SCC in STR 2 L Fg: 1 vvm Turbine impeller 450 rpm 25 ± 2 °C 16/8 h photoperiod (140 µmol m^−2^ s^−1^)	330 g/L	220 g/L	Anthroquinone production was 2.5 times higher in STR	[125]
*Arnebia* sp.	Shikonin	25 mL CSC in 250 mL flasks	Air-lift bioreactor	1249.2 g/L	480 g/L	The shikonin content was 2.6 times higher in the bioreactor than in the flask. Production remained without significant differences in both bioreactors	[126]
		100 rpm	2 L working volume				
		25 ± 2 °C	25 ± 2 °C				
		Continuous light	Fg: 2 L/min (sparger ring)				
		(70 µmol/m^2^ s ^1^)					
			STR 2 L	1249.2 g/L	450 g/L		
			Six-blade turbine impeller 100 rpm				
			Fg: 2 L/min				
			25 ± 2 °C				
*Ocinum basilicum*	Rosmarinic acid	100 rpm	7 L CSC in STR 10 L	Biomass was 8.4 times higher in bioreactor than in flask		Production increased 1.66 times in bioreactor	[69]
		25 ± 2 °C	Marine impeller 100 rpm				
			Fg: 25 L/min				
*Satureja khuzistanica*	Rosmarinic acid	200 mL CSC in 1 L flask	1 L CSC in culture bags 2 L	13.6 g/L	18.7 g/L	Production increased 2.5 times in bioreactor	[127]
		110 rpm	Batch mode				
		25 °C	20–30 rpm				
			25 °C				
			Fg: 0.1 vvm				
			Darkness				
*Vitis labrusca* L.	Resveratrol	100 mL CSC in 300 mL flasks	STR 5 L	NR	≈35 g DW	Production increased 1.15 times in bioreactor	[128]
		110 rpm	Marine impeller 110 rpm				
		23 °C	Fg: 0.15 vvm				
		Darkness					
*Santalum album* L.	Squalene	100 mL CSC in 250 L flask	Airlift bioreactor 7 L	1.05 mg/g	1.25 mg/g	Production increased 1.71 times in bioreactor in four weeks of culture	[129]
		90 rpm	Batch mode				
		28 °C	70–80 rpm				
			Fg: 4 L/min				
			28 ± 2 ° C				

NR means Not reported.

**Table 6:** Models used to describe kinetics and deactivation in whole cells [137,140,141,142].

Mathematical Equation	Conventional Name
$\begin{array}{l} r_{x}=µ=\frac{{µ}_{\max}[S_{i}]}{[S_{i}]+K_{m}}\\ r_{s}=Y_{\mathrm{xs}}µ \end{array}$	Monod kinetics
$\begin{array}{l} r_{x}=µ=\frac{{µ}_{\max}[S_{i}]}{({\left[S_{i}\right]}^{2}/K_{i})+[S_{i}]+K_{m}}\\ r_{s}=Y_{\mathrm{xs}}µ \end{array}$	Expanded Monod kinetics
$\begin{array}{l} r_{x}=µ=\frac{{µ}_{\max}[S_{i}]}{[S_{i}]+K_{m}}\left(1-\frac{\left[P\right]}{{\left[P\right]}_{\max}}\right)\\ r_{s}=Y_{\mathrm{xs}}µ \end{array}$	Expanded Monod kinetics
$\begin{array}{l} r_{x}=µ={µ}_{\max}(1-\exp(-[S_{i}]/K_{m}))\\ r_{s}=Y_{\mathrm{xs}}µ \end{array}$	Monod’s teacher Tessier kinetics.
$\begin{array}{l} r_{x}=µ=\frac{{µ}_{\max}[S_{i}]}{[S_{i}]+K_{S}X}\\ r_{s}=Y_{\mathrm{xs}}µ \end{array}$	Contois kinetics.
$\begin{array}{l} r_{x}=µ={µ}_{\max}\left(1-\frac{X}{K_{S}}\right)\\ r_{s}=Y_{\mathrm{xs}}µ \end{array}$	Logistic kinetics.
$\begin{array}{l} \frac{d\theta_{x}}{dt}=-k_{\mathrm{in}}{\left(\theta_{x}-\theta_{ss}\right)}^{m}\\ r=r_{s}=\theta_{x}\frac{k[S_{i}]}{[S_{i}]+K_{m}} \end{array}$	Cell deactivation kinetics

## Abbreviations

k_in_	deactivation constant.
K_m_	affinity constant.
K_s_	substrate inhibition constant.
m	deactivation order.
n	reaction order value affecting the concentration of substrate and affinity constant.
r and r_s_	the specific consumption rate of substrate i.
r_x_	the specific rate of biomass formation.
r_max_	the maximum specific consumption rate of substrate i.
[S_i_]	the concentration of substrate i.
X	the concentration of biomass.
Y_x/s_	biomass yield from substrate.
µ	the specific rate of biomass formation.
µ_max_	the maximum specific rate of biomass formation
θ_X_	dimensionless cell deactivation.
θ_ss_	residual dimensionless activity.
